# The Cytoskeletal Protein Cyclase-Associated Protein 1 (CAP1) in Breast Cancer: Context-Dependent Roles in Both the Invasiveness and Proliferation of Cancer Cells and Underlying Cell Signals

**DOI:** 10.3390/ijms20112653

**Published:** 2019-05-30

**Authors:** Rokib Hasan, Guo-Lei Zhou

**Affiliations:** 1 Molecular Biosciences Graduate Program, Arkansas State University, State University, AR 72467, USA; mdrokib.hasan@smail.astate.edu; 2 Department of Biological Sciences, Arkansas State University, State University, AR 72467, USA

**Keywords:** breast cancer, CAP1, the actin cytoskeleton, cell adhesion, cell invasiveness, cell proliferation, ERK, FAK

## Abstract

As a conserved actin-regulating protein, CAP (adenylyl Cyclase-Associated Protein) functions to facilitate the rearrangement of the actin cytoskeleton. The ubiquitously expressed isoform CAP1 drives mammalian cell migration, and accordingly, most studies on the involvement of CAP1 in human cancers have largely been based on the rationale that up-regulated CAP1 will stimulate cancer cell migration and invasiveness. While findings from some studies reported so far support this case, lines of evidence largely from our recent studies point to a more complex and profound role for CAP1 in the invasiveness of cancer cells, where the potential activation of cell adhesion signaling is believed to play a key role. Moreover, CAP1 was also found to control proliferation in breast cancer cells, through the regulation of ERK (External signal-Regulated Kinase). Alterations in the activities of FAK (Focal Adhesion Kinase) and ERK from CAP1 depletion that are consistent to the opposite adhesion and proliferation phenotypes were detected in the metastatic and non-metastatic breast cancer cells. In this review, we begin with the overview of the literature on CAP, by highlighting the molecular functions of mammalian CAP1 in regulating the actin cytoskeleton and cell adhesion. We will next discuss the role of the FAK/ERK axis, and possibly Rap1, in mediating CAP1 signals to control breast cancer cell adhesion, invasiveness, and proliferation, largely based on our latest findings. Finally, we will discuss the relevance of these novel mechanistic insights to ultimately realizing the translational potential of CAP1 in targeted therapeutics for breast cancer.

## 1. CAP1 as a Versatile Actin-Regulating Protein That Promotes Actin Filament Turnover

### 1.1. CAP, Identified as a Protein That Interacts with Adenylyl Cyclase in Yeast, Is a Conserved Actin-Regulating Protein across Eukaryotes

CAP was first identified in the budding yeast *S. cerevisiae* three decades ago. Two groups simultaneously reported it, and named the protein as CAP [1] and SRV2 (Suppressor of the activated RAS2^Val-19^ allele) [2], respectively. Distinct approaches were employed with yet a somewhat shared focus on its biochemical and functional interactions with adenylyl cyclase, which mediates the regulation of the enzyme via the RAS onco-protein. The first study identified yeast CAP as a protein that associates with adenylyl cyclase, and the N-terminus domain of CAP was later found to be responsible for the interaction with adenylyl cyclase, and it is required for RAS to activate the enzyme [3,4,5]. The other study found that perturbation of the *CAP* gene in budding yeast causes the cells to fail to respond to the activated RAS2^Val-19^ [2]. Notably, while the name CAP has been used predominantly, the biochemical or functional interactions between CAP and adenylyl cyclase has only been confirmed in *S. cerevisiae*, the fission yeast *S. pombe* [6], *C. ablicans* [7], and Dictyostelium [8]. In higher eukaryotes including mammals, there is a lack of solid evidence supporting a conservation of this CAP/adenylyl cyclase interaction; in fact, the adenylyl cyclase structure has not been conserved during evolution, and the N-terminus domain of CAP that mediates the interaction with adenylyl cyclase has the least conserved amino acid sequence among the structural domains on CAP homologues. 

Besides the phenotypes related to the defected RAS signaling, yeast cells with *CAP* deletion also exhibit changes in morphology, as cells are larger and rounder, suggesting a disrupted cytoskeletal structure. Further studies reveal that both yeast CAP homologues are bifunctional proteins, where its N-terminus mediates RAS/cAMP signaling, while the C-terminus binds and sequesters monomeric actin (G-actin) to regulate the actin cytoskeleton [6,9,10]. In maintaining the structure of the actin cytoskeleton and facilitating its dynamic rearrangement, monomeric actin is continuously polymerized into one end of actin filaments, while being released from the other end [11]. Sequestering actin monomers is a shared capacity of actin-binding proteins, which enables cells to maintain a pool of actin monomers that is at a much higher concentration than the critical concentration at which the monomers will polymerize into filaments in vitro [12]. This actin monomer-sequestering ability was initially believed to be solely responsible for the function of CAP in the actin cytoskeleton. Lines of evidence from the rescue of the actin cytoskeletal phenotypes in the yeast cells with the deletion of *CAP* gene by CAP homologues from other species, as well as the phenotypes observed in organisms with CAP1 perturbation, consistently support a conserved function for CAP in regulating the actin cytoskeleton and actin-dependent cell functions. These species include Dictyostelium, fungi, Drosophila, *C. elegans*, plants, and mammals [7,13,14,15,16,17,18,19,20,21,22,23,24,25]. Mammals have two isoforms, CAP1 and CAP2, which have considerably diverged amino acid sequences [16]. CAP1 is ubiquitously expressed, and it has been more intensively studied, while the expression of CAP2 is limited to a few specific tissue types [26]. The main focuses of CAP studies have been on the roles and mechanisms for yeast and mammalian CAP homologues in regulating actin dynamics and actin-dependent functions. Two reviews on CAP have provided detailed overview on these aspects [27,28], and the fact that these reviews were published a decade apart from each other also helps readers grasp a comprehension of the progression of studies on CAP. 

### 1.2. CAP Promotes Actin Filament Turnover through Multiple Mechanisms That Are Mediated by All of Its Structural Domains

**Figure 1:**
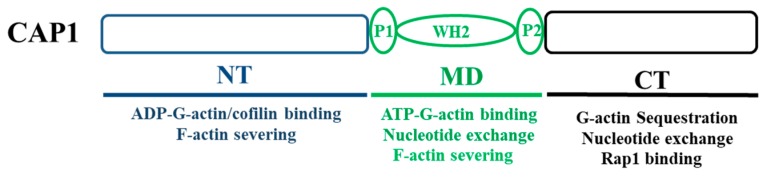
The structural organization of CAP1. CAP1 can be divided into three domains that are conserved across CAP homologues from species. Highlighted are the functions that are mediated by separate domains that are relevant to cell migration, largely for those important in facilitating actin dynamics. The C-terminus also binds Rap1, which can may mediate CAP1 functions in cell adhesion and proliferation. CAP1 also associates with FAK/talin to regulate those cell functions; however, the domain on CAP1 responsible for this interaction has yet to be determined, and are thus not shown here.

While binding and sequestering G-actin through its C-terminus domain was initially thought to be the mechanism that was solely responsible for the CAP function in the actin cytoskeleton, consequent studies, primarily on yeast and mammalian homologues, reveal that CAP facilitates actin dynamics through much more versatile roles, as executed by its separate structural domains. Virtually all CAP proteins consist of three structural domains: the N-terminus domain, a highly conserved C-terminus domain, and a well conserved and proline-rich middle domain that also harbors a WH2 (WASP Homology domain 2) domain [28,29,30]. Further studies reveal that all three domains play important roles in facilitating all key steps in the cycle of actin filament turnover. It was first found that each domain of mammalian CAP1 executes separate functions in promoting cofilin-driven actin filament turnover [31]. These include the depolymerization of actin filaments, and the relief of the inhibitory effect of cofilin on nucleotide exchange for ADP-G-actin, as ADP-G-actin must be recharged to become ATP-G-actin before it can be added onto the barbed end of filaments for polymerization [31]. Moreover, the N-terminus of yeast CAP also interacts with the cofilin-actin complex, to accelerate cofilin-mediated actin filament turnover [32]. Furthermore, the WH2 domain of yeast CAP was found to play a central role in recharging the ADP-G-actin, while in the context of the intact protein [29]. In contrast, the C-terminus β-sheet domain was essential and sufficient for mouse CAP1 to catalyze the nucleotide exchange, and the adjacent WH2 domain is not required for this function [33]. Finally, CAP1 was also found to act directly on actin filaments to accelerate cofilin-mediated actin severing [34]. At a neutral pH that is characteristic of that in the cytosol in mammalian cells, neither CAP1 nor cofilin is capable of effectively severing actin filaments. In contrast, the combination of CAP1 and cofilin can rapidly sever actin filaments at all pH values across the physiological range. In summary, CAP regulates actin dynamics through its highly versatile functions, by facilitating all key steps in the cycle of actin filament turnover, as mediated by its separate structural domains. Figure 1 shows the domain structure of CAP1, which is conserved in virtually all homologues, highlighting the roles and binding partners that are important for the protein to facilitate actin dynamics and regulate cell migration. The above findings further support CAP as a key regulator of the actin cytoskeleton, and actin-dependent cell functions, and further, relevant biological and pathological processes, such as cancer metastasis. 

### 1.3. Loss of CAP1 Functions Universally Leads to Enhanced Stress Fibers in Mammalian Cells

A loss of CAP function in yeast cells leads to a disrupted actin cytoskeleton, consistent with its cellular function as an actin cytoskeletal protein. In the mammalian system, which is more relevant to the focus of this review, enhanced actin stress fibers have been consistently observed in CAP1-knockdown cells [35,36,37]. This phenotype is expected, and it is believed to derive from the loss of CAP1 functions in both sequestering the actin monomers that would promote actin polymerization, and in promoting actin filament turnover [38]. Actin stress fibers, along with other two subcellular structures that are rich in filamentous actin in filopodia and lamellipodia, play roles in cell movement by contributing to cell adhesion [39], and they are believed to also transduce the tension force required for detaching the trailing edge of the cell so that the cell body can be pulled forward during migration. However, under some conditions, stress fibers can instead inhibit cell motility [39]. The abnormally enhanced stress fibers probably exert a similar role to that of cell adhesion that is too strong; while cell adhesion generates the traction force that is essential for cell movement, adhesion that is too strong actually hinders cell migration, since the cell will have difficulty in overcoming it, for the cell to migrate. The reduced actin filament turnover, as suggested by the enhanced stress fibers, at least partially explain why this phenotype is associated with a reduced rate of cell migration. The repeated cycles of actin filament turnover drives cell movement, also by promoting the dynamic formation and turnover of the important pro-migratory structures, filopodia and lamellipodia. Based on these, the presumption was that up-regulated expression levels of CAP1 would stimulate cell motility and cancer cell invasiveness. However, evidence is accumulating against universal up-regulation of CAP1 in cancer cells, and suggests that CAP1 plays more complex roles in cell migration and cancer cell invasiveness, where the involvement of CAP1 in cell adhesion is likely to be critical.

## 2. Cell Context-Dependent Roles for CAP1 in Cell Migration, and the Invasiveness of Human Cancers

Studies in our group and others have solidly established roles for CAP1 in regulating the actin cytoskeleton and migration in mammalian cells [31,35,36]. Moreover, we have unraveled cell context-dependent roles for CAP1 in cell motility and cancer cell invasiveness [36,40]. We first found a new function for CAP1 in regulating adhesion of HeLa cells, by also revealing biochemical and functional interactions between CAP1 and the adhesion kinase FAK, and talin as well [36]. Knockdown of CAP1 in HeLa cells unexpectedly led to activation of FAK and enhanced cell adhesion, which is believed to have overcome the negative effect on cell migration from the reduced rate of actin filament turnover; collectively, the depletion of CAP1 actually stimulated cell motility and invasiveness [36]. Importantly, our follow-up study supports the conservation of this functional interaction between CAP1 and FAK in breast cancer cells, where CAP1 actually fulfills more complex roles in regulating FAK, and cell adhesion and invasiveness, depending on the cell type [40]. Depletion of CAP1 led to opposite alterations in FAK activity and phenotypes in the adhesion and invasiveness in the metastatic and non-metastatic breast cancer cells, with the metastatic cancer cells behaving similarly to HeLa cells. Interestingly, supporting our identification of the role for CAP1 in regulating cell adhesion and proliferation, CAP1 was recently found to bind the G-protein Rap1 [41], a known regulator of cell proliferation, as well as adhesion by modulating integrin affinity or activation [42,43]. Besides promoting the formation and turnover of focal adhesions [44], activated FAK can also stimulate the formation of lamellipodia [45,46]. Therefore, while the knockdown of CAP1 in HeLa and metastatic breast cancer cells reduces the rate of actin filament turnover, it causes the activation of FAK, and possibly also altered Rap1 signaling, which not only promotes cell adhesion and turnover of focal adhesions, but may also promote lamellipodia formation to promote cell motility and invasiveness. Lastly, we also detected the elevated activity of ERK in the CAP1-knockdown metastatic breast cancer cells, while reduced ERK activity was detected in the non-metastatic cancer cells [40]. ERK is best known as a key regulator of cell proliferation that drives cell cycle through the up-regulation of Cyclin D1 levels [47,48]; meanwhile, it can also stimulate cancer cell invasiveness through the Snail/E-Cadherin axis [49,50]. Consistent with the context-dependent roles in cell motility and cancer cell invasiveness, mounting evidence argues against a scenario where CAP1 is up-regulated in cancer such that it would universally stimulate cancer cell invasiveness. In addition to our recent findings, as discussed above, the available data from a high-throughput screening suggests that only colon cancer had a considerable degree of up-regulation of CAP1, compared to that in the non-cancer tissues, out of the 20 cancer types examined (http://www.proteinatlas.org/ENSG00000131236-CAP1/cancer). To date, CAP1 has been implicated largely in the invasiveness, of a growing list of human cancers, including breast, lung, pancreatic, and ovarian cancer, as well as neural glioma, hepatocellular carcinoma, and head and neck squamous cell carcinomas [40,51,52,53,54,55,56,57,58,59,60,61,62]. On the other hand, the other mammalian isoform, CAP2, is implicated in hepatocellular carcinoma and melanoma [63,64,65]. It is noted, however, that while the case for the involvement of CAP in human cancers is increasingly better established, some inconsistency remains in terms of the roles of the proteins, and the underlying mechanisms remain insufficiently understood.

## 3. A Novel Function for CAP1 in Regulating ERK and the Proliferation of Breast Cancer Cells

As a cytoskeletal protein, CAP1 was not originally expected to play a role in the proliferative transformation of cancer cells, and such a role had rarely been explored. We previously reported that the mitochondrial shuttling of CAP1 was involved in the apoptosis of HeLa cells, through the intrinsic apoptotic pathway [66]; however, this function was largely attributed to the role of CAP1 as a component of the actin cytoskeleton. The fact that the depletion of CAP1 led to altered ERK activity suggests that CAP1 may actually play a role in the proliferation of breast cancer cells, which promoted us to test it. Indeed, we detected cell proliferation phenotypes that were consistent with the altered ERK activity in both the CAP1-knockdown metastatic and non-metastatic breast cancer cells [40]. Similar to the case with effects on FAK activity derived from CAP1 depletion, opposite effects on ERK activity were detected in the metastatic and non-metastatic breast cancer cells, where elevated ERK activity in the metastatic breast cancer cells was detected along with activated FAK. We also detected increased anchorage-independent growth in the CAP1-knockdown metastatic breast cancer cells [40]. Therefore, in addition to its traditionally focused roles in the actin cytoskeleton and cancer cell invasiveness, CAP1 also regulates cancer cell proliferation through ERK, at least in breast cancer. It is possible that Rap1 may also be involved in mediating CAP1 signals to regulate proliferation. Together, these findings support roles for CAP1 in both the morphological and proliferative transformation processes, which are the two most prominent hallmarks of cancer [67]. The signaling kinases FAK and ERK likely play central roles, by mediating CAP1 signals to control the key cancer cell functions, including adhesion, invasiveness, and proliferation. 

## 4. A Signaling Molecule That Links CAP1 to the Regulation of ERK

Altered ERK activities at least partially underlie the cell context-dependent roles of CAP1 in the proliferation of breast cancer cells, while likely also contribute to the phenotypes in cancer cell invasiveness. The yeast CAP homologues were found to be bifunctional proteins involved in both cell signaling and the regulation of the actin cytoskeleton, the fact that CAP harbors the SH3 (Src Homology 3) binding site, and the identification of interactions with a number of signaling molecules [68,69,70], provided supporting evidence for a further dimension of involvement of CAP in cell signaling. However, the pertinent literature does not support a direct interaction between CAP1 and ERK, and rather, another molecule(s) likely functions to link these two together. In this regard, the above mentioned G-protein Rap1 as well as two tyrosine kinases, Abl and FAK, emerged as possible candidate signaling molecules, considering that all of them interact with CAP1 and also regulate cell adhesion and/or proliferation, including that through ERK. Abl can activate ERK through Rap1 and B-Raf [71], and it has also been reported that the SH3 domain of human Abl binds human CAP1 through the proline-rich middle domain [72]. The Drosophila homologue of CAP functionally collaborates with Abl in controlling the midline axon pathfinding downstream of multiple Roundabout receptors [70]. Therefore, a potential conservation of this interaction in breast cancer cells may explain that the depletion of CAP1 causes the activation of Abl, and consequently that of ERK. On the other hand, while FAK is best known to regulate cell adhesion, it has also been reported that the activated FAK/integrin signaling activates ERK [73,74]. It is therefore possible that FAK, which is indeed activated in the CAP1-knockdown metastatic breast cancer cells [40], was actually responsible for ERK activation. 

**Figure 2:**
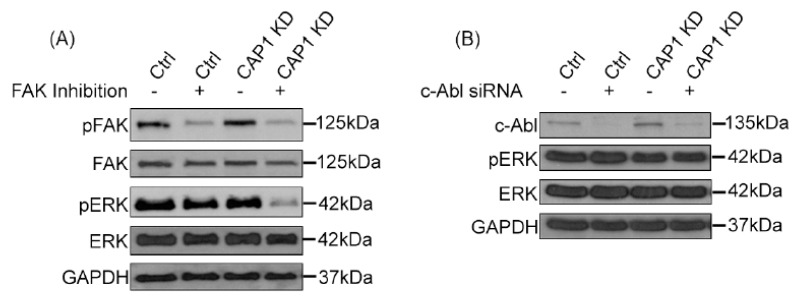
Inhibition of FAK activity, but not the depletion of c-Abl, reduced ERK activity in MDA-MB-231 cells, suggesting that FAK mediates CAP1 signals to regulate ERK. (**A**) The control and CAP1-knockdown MDA-MB-231 metastatic breast cancer cells were treated with 5 μg/mL FAK inhibitor PF-573228 (SelleckChem) for 13 h before cells were harvested for Western blotting, to detect the effects on ERK activity. Antibodies against FAK, pFAK(Tyr397), ERK, and pERK (Thr202/Tyr204) were from Cell Signaling Technology Inc., and that against GAPDH (Glyceraldehyde-3-Phosphate Dehydrogenase) was from Santa Cruz Biotechnology Inc. The control indicates the stable cells that harbor the empty vector of the CAP1 knockdown shRNA construct, and CAP1KD indicates CAP1-knockdown-stable cells that we previously established. (**B**) Transient silencing of c-Abl with the siRNA (SCBT #sc-29310) in the control and CAP1-knockdown MDA-MB-231 cells did not have a remarkable effect on ERK activity. The antibody against c-Abl was from Cell Signaling Technology Inc. Cells were cultured for 24 h in DMEM supplemented with 10% fetal bovine serum and then transfected with the siRNA, following the manufacturer’s protocol. Cells were harvested 48 h after transfection for analyses in Western blotting, to confirm the depletion of c-Abl and its effects on ERK activity.

**Figure 3:**
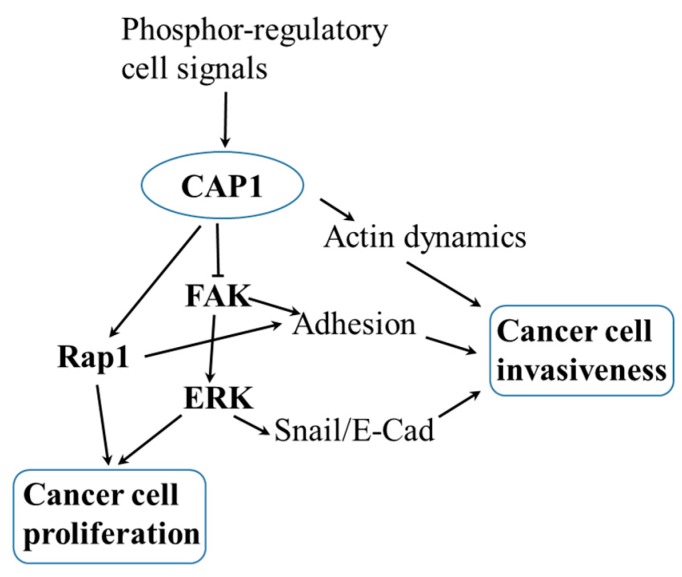
A schematic model depicting how CAP1 likely regulates the FAK/ERK axis and Rap1, and through these, controls invasiveness and proliferation in metastatic breast cancer cells. Knockdown of CAP1 activates FAK, and which in turn activates ERK. The activated ERK and Rap1 lead to elevated cell proliferation. On the other hand, enhanced cell adhesion and increased turnover of focal adhesions from activated FAK and Rap1, as well as the activated ERK/Snail/E-Cadherin signals may collectively stimulate cancer cell invasiveness, and overcome negative effects on cancer cell invasiveness from the reduced rate of actin filament turnover, due to the loss of CAP1 function in these cells. The cell signals that function in cohort to control transient phosphorylation at the S307/S309 tandem regulatory site on CAP1 likely control the cancer cell functions through CAP1.

We next tested if FAK or Abl indeed fulfills the role in linking CAP1 to ERK, by manipulating FAK or Abl signaling in the MDA-MB-231 cells, followed by determining their effects on ERK activity. Firstly, we employed the approach of chemical inhibition of the kinase activity of FAK or Abl. As shown in Figure 2A, treatment with the FAK inhibitor PF-573228 effectively reduced FAK activity in both the control and CAP1-knockdown cells, as detected in Western blotting using the phosphor FAK antibody against Y397. Interestingly, we detected remarkably decreased ERK activity in these cells as well, using the phosphor-specific antibody against Thr202/Tyr204 on ERK (Figure 2A). These results suggest that elevated FAK activity is at least partially responsible for causing the activation of ERK in the metastatic breast cancer cells. In testing the potential role of Abl, the phosphor-specific antibody against c-Abl did not perform well enough for the results to be conclusive. We thus alternatively employed the approach of transient knockdown of Abl with lentiviral-based siRNA. As shown in Figure 2B, the siRNA effectively reduced the expressional levels of Abl 48 hrs after the transfection. However, the depletion of Abl did not cause any remarkable change in the activity of ERK. Taken together, these results support that FAK is responsible, at least partially, for mediating CAP1 signals to regulate ERK in the metastatic breast cancer cells. Thus, the signaling axis of FAK/ERK is responsible to function downstream of CAP1 to mediate its signals to control cell adhesion, invasiveness as well as proliferation. Whereas our results do not support such as role for Abl, we cannot totally exclude involvement of the Abl family kinases, since the siRNA did not target another member Arg (*ABL*-related gene). It is also noted that Rap1 may also play a role in mediating CAP1 signals to regulate these cell functions. Figure 3 shows the schematic model depicting how the FAK/ERK axis, and possibly the Rap1 signaling as well, likely functions downstream of CAP1 to regulate the actin dynamics, cell adhesion, and Snail/E-Cadherin to control both the invasiveness and proliferation of breast cancer cells. 

## 5. Molecular Mechanisms That May Underlie the Regulation of CAP1 Function in Cell Adhesion

Our findings support that the role for CAP1 in cell adhesion is critical in the cell context-dependent roles for CAP1 in cancer cell invasiveness, which is highly relevant to cancer metastasis that accounts for the death of most cancer patients. FAK and Rap1 likely both mediate CAP1 signals to regulate cell adhesion. As we previously discussed [38], a likely mechanism for the depletion of CAP1 to activate FAK is that it releases talin and thus allows more talin molecules to bind to the cytoplasmic tails of integrins, which triggers the inside-out signaling [75] leading to the activation of integrins and FAK, and enhanced cell adhesion [38]. Alternatively, the association with CAP1 may directly inhibit the activity of FAK independent of integrins. In this scenario, depletion of CAP1 would relieve this inhibition and subsequently, it lead to FAK activation. However, in either case, it is intriguing how the depletion of CAP1 in the metastatic and non-metastatic breast cancer cells can actually exert opposite effects on FAK activity and cell adhesion [40]. Unravelling whether CAP1 interacts differentially with the adhesion molecules (FAK, Rap1, and talin) involved in these cell types may shed light on the underlying molecular mechanism.

Recent findings from our group support that phosphorylation of CAP1 also regulates its function in cell adhesion. Neither the phosphor-mimetic (S307D/S309D) nor the unphosphorylatable (S307A/S309A) mutants of CAP1, which resist regulation through transient phosphorylation, were able to rescue the elevated FAK activity or the enhanced focal adhesion phenotypes in the CAP1-knockdown HeLa cells, whereas the re-expressed wild type CAP1 effectively alleviated these phenotypes. It is possible that the phosphorylation of CAP1, which controls the association/dissociation of CAP1 from cofilin and actin [37], may also regulate its interactions with FAK and Rap1. In the case of FAK, elevated CAP1 phosphorylation at the S307/S309 tandem site was detected in cells cultured in suspension [37], in which reduced FAK activity is expected. Thus, a reasonable scenario would be that the phosphorylation on CAP1 enhances CAP1 association with FAK, which inhibits the kinase activity of FAK. 

## 6. Relevance of These Mechanistic Insights to Realizing the Translational Potential of CAP1 in Breast Cancer 

Mounting evidence suggests the involvement of CAP1 in a growing list of human cancers, with emerging evidence suggesting a more complex role that is dependent on the type (or even the subtype) of cancer. We unraveled cell context-dependent roles for CAP1 in regulating the invasiveness of breast cancer cells, as well as a role in controlling the proliferation of cancer cells. Moreover, we unraveled that ERK likely plays a key role in mediating CAP1 signals to control both cell functions. Studies on CAP1 in our group over the last several years have led to the identification of a new cellular function for the protein in cell adhesion, its very first regulation mechanism through phosphorylation, as well as the profound and complex roles in the key functions of breast cancer cells [36,37,40]. We also found that phosphor-regulation at the S307/S309 tandem site plays important roles in the proliferation and invasiveness of breast and pancreatic cancer cells [40,62]. These mechanistic insights will surely help us better to understand the somewhat unexpected context-dependent roles for CAP1 in the invasiveness and proliferation of breast cancer cells.

Our findings that the FAK/ERK axis mediates CAP1 signals represent an important contribution to the knowledge on how CAP1 controls both invasiveness and proliferation in breast cancer cells. Along with findings from other studies that also support the involvement of CAP1 in breast cancer cell functions, they carry important implications in targeted therapeutics for the disease. Firstly, more complex roles for CAP1 in cell migration and invasiveness that also involve cancer cell adhesion have led to a new concept of how CAP1 may control the invasiveness of breast cancer. Secondly, the phosphor-regulation mechanism of CAP1, as well as the responsible phosphor-regulatory cell signals that are identified, may help to develop strategies for targeting CAP1 in breast cancer therapeutics without directly targeting the cytoskeletal protein. De-regulated cell signal transduction has been widely pursued as a therapeutic target for cancer treatment, while targeting the cytoskeleton itself or its components is in general less feasible and not well-tolerated. Therefore, these findings may open up avenues targeting the upstream phosphor-regulatory signals of CAP1, as well as the downstream cell signals that mediate CAP1 signals to control cancerous transformation and metastatic progression, such as ERK and FAK. Lastly, breast cancer is a highly diverse disease where thousands of genes may contribute to its pathophysiologies, and the genomic and transcriptional characteristics of 51 breast cancer cell lines mirror those of 145 breast cancer tumors [76]. The cell context-dependent roles for CAP1 in breast cancer suggests that developing a targeting strategy tailored for the specific subtype of breast cancer can be critical in order to achieve desired treatment outcomes in targeting CAP1 or related cell signals in the treatment of breast cancer. 

Enhanced invasiveness and uncontrolled proliferation of cancer cells underlie the morphological and proliferative transformations, respectively, which are arguably the two most prominent hallmarks of cancer cells. The goal in targeted cancer therapeutics is to suppress these de-regulated cell functions, resulting in apoptosis and the control of the invasive cycle of cancer cells. Since CAP1 can function to either stimulate or suppress these cell functions, depending on the subtype of breast cancer that originates from these distinct cancer cell types, considerations should be taken for developing strategies that targeting the upstream regulatory cell signals of CAP1, to either enhance or inhibit CAP1 function.

## 7. Concluding Remarks

Mammalian CAP1 has been the primary focus of CAP studies over the last decade and a half, in large part due to its emerging biomedical implications. Studies from our group and others have solidly established roles for CAP1 in regulating cell functions, the de-regulation of which underlies the major hallmarks of cancer, such as the actin cytoskeleton, cell adhesion, migration, and proliferation [35,36,40]. Our studies unravel profound and complex roles for CAP1, involving key functions including adhesion, invasiveness, and proliferation in breast cancer cells. It remains to be better determined whether such roles of CAP1 are conserved across other cancer types. Regardless, these mechanistic insights help to shape a more complete and in-depth picture concerning the roles of CAP1 in human cancers, such insights are also imperative before realizing that the translational potential of CAP1 can be possible.

In summary, we found that CAP1 not only controls breast cancer cell invasiveness, which was more of an anticipated one, but it also regulates cancer cell proliferation. Moreover, the FAK/ERK axis appears to play a critical role in mediating CAP1 signals, to regulate cancer cell functions, the de-regulation of which underlie both morphological and proliferative transformations. Finally, we demonstrate opposite roles for CAP1 in metastatic and non-metastatic cancer cells. The identified cell-signaling molecules and pathways that are involved in regulating CAP1, as well as mediating CAP1 signals, including GSK3, FAK, and ERK, may be therapeutic targets in novel treatment strategies for breast cancer and potentially other cancer types, especially from the perspective of targeting CAP1-related cell signals. Indeed, consistent with their key roles in cell functions underlying human cancers, GSK3, FAK, and ERK have all been proposed and pursued as targets of intervention in cancer therapeutics [77,78,79,80,81,82].

## Abbreviations

CAP1	adenylyl Cyclase-Associated Protein
ERK	External signal-Regulated Kinase
FAK	Focal Adhesion Kinase
GSK3	Glycogen Synthase Kinase 3
SRV2	Suppressor of the activated RAS2^Val-19^ allele
WH2	WASP Homology domain 2
SH3	Src Homology 3
Rap1	Ras-related protein 1
